# Clinical Outcomes of Sequential Intrastromal Corneal Ring Segments and an Extended Range of Vision Intraocular Lens Implantation in Patients with Keratoconus and Cataract

**DOI:** 10.1155/2018/8328134

**Published:** 2018-04-19

**Authors:** C. Lisa, R. Zaldivar, A. Fernández-Vega Cueto, R. M. Sanchez-Avila, D. Madrid-Costa, J. F. Alfonso

**Affiliations:** ^1^Fernández-Vega Ophthalmological Institute, Oviedo, Spain; ^2^Instituto Zaldivar, Mendoza, Argentina; ^3^Centro de Oftalmología Barraquer, Barcelona, Spain; ^4^Optics II Department, Faculty of Optics and Optometry, Universidad Complutense de Madrid, Madrid, Spain

## Abstract

**Purpose:**

To evaluate efficacy, safety, and predictability of sequential Ferrara-type intrastromal corneal ring segments (ICRS) and an extended range of vision intraocular lens (IOL) implantation in patients with keratoconus and cataract.

**Methods:**

This study comprised patients with keratoconus and cataract that had ICRS implantation followed 6 months later by extended range of vision IOL implantation. The uncorrected distance visual acuity (UDVA), corrected distance visual acuity (CDVA), and residual refractive errors, analysed using vector analysis, were recorded preoperatively, 6 months after ICRS implantation, and 6 months after IOL implantation, respectively.

**Results:**

The study enrolled 17 eyes (11 patients). The mean UDVA (logMAR scale) was 1.15 ± 0.67 preoperatively, 0.88 ± 0.69 six months after ICRS implantation (*P* = 0.005), and 0.27 ± 0.18 six months after IOL implantation (*P* < 0.0001). The CDVA changed from 0.26 ± 0.15 (logMAR) before surgery to 0.17 ± 0.08 six months after Ferrara-type ICRS implantation (*P* = 0.002) and to 0.07 ± 0.06 six months after IOL implantation (*P* < 0.0001). The spherical equivalent and the refractive cylinder declined steeply after IOL implantation (*P* < 0.001). The magnitude of depth of focus was 2.60 ± 1.02 D. There were no statistically significant differences in visual acuity for a defocus range from +0.50 D to −0.50 D (*P* > 0.1).

**Conclusion:**

Sequential Ferrara-type ICRS and an extended range of vision IOL implantation provided good visual and refractive outcomes, being an effective, safe, and predictable procedure for the treatment of selected cases of patients with keratoconus and cataract. In addition, this approach provides an increase of tolerance to defocus.

## 1. Introduction

The most common human ocular afflictions are presbyopia and cataract [1]. Both presbyopia and cataract developments contribute to further decreased visual quality of keratoconic patients. Furthermore, it has been suggested that patients affected by keratoconus tend to develop cataracts sooner than others [2]. Several options have been proposed for replacement of the lens (either by refractive lens exchange or cataract removal). Toric intraocular lens (IOL) implantation has shown to be an effective and safe option to improve the uncorrected distance visual acuity (UDVA), corrected distance visual acuity (CDVA), and refractive error [3–10]. Multifocal toric IOL implantation has shown encouraging outcomes [11–13]. The main problem for these IOLs is that the corneal irregularities are still present after IOL implantation, and it could restrict the visual rehabilitation. In fact, it has been reported that keratoconic patients with more regular corneas obtained higher improvement in UDVA after cataract surgery and toric IOL implantation [8]. Another important challenge is the IOL power calculation. A combined procedure, instrastromal corneal ring segments (ICRS) implantation followed by cataract surgery with IOL implantation, has also been proposed [14, 15]. This approach could have a double benefit. By one way, ICRS implantation improves the corneal shape and consequently the visual quality; on the other hand, improving the corneal shape could help the IOL estimation [14]. Basing on this previous experience, we currently present a case series of patients affected by cataract and keratoconus who underwent ICRS implantation followed by an extended range of vision IOL implantation. By means of increasing the depth of focus, this approach has a two-fold objective: one is to improve the visual acuity from far to intermediate distances and the other is to increase the tolerance to defocus, making the IOL calculation a little less important.

## 2. Patients and Methods

This study was a retrospective longitudinal analysis of the visual and refractive results of sequential implantation of the Ferrara-type ICRS (AJL Ophthalmic, Spain) and an extended range of vision IOL implantation in eyes with keratoconus and cataract. It was carried out at Fernández-Vega Ophthalmological Institute, Oviedo, Spain. The tenets of the Declaration of Helsinki were followed, and full ethical approval from the institute was obtained. After receiving a full explanation of the nature and possible consequences of the study and surgery, all patients signed the informed consent.

The presence of keratoconus and cataract, contact lens intolerance, and a clear cornea, along with a minimum corneal thickness over 400 *μ*m at the optical zone involved in the implantation (a general criterion for surgery), constituted the criteria for inclusion in the study. In addition, the keratoconus had to be stage I, II, or III according to the Amsler-Krumeich keratoconus classification. Keratoconus was diagnosed by combining computerised videokeratography of the anterior and posterior corneal surfaces (Sirius, CSO, Italy), K readings, and corneal pachymetry [16–18]. Contact lens use was discontinued 1 month prior to corneal topography.

The exclusion criteria defined for the study were previous corneal or intraocular surgery, history of herpetic keratitis, diagnosed autoimmune disease, systemic connective tissue disease, endothelial cell density < 2000 cells/mm^2^, history of glaucoma or retinal detachment, macular degeneration or retinopathy, neuroophthalmic diseases, and history of ocular inflammation.

All eyes in this study received Ferrara-type ICRS (AJL Ophthalmic, Spain). These Ferrara-type ICRS are poly(methyl methacrylate) with a triangular cross section that induces a prismatic effect on the cornea. The apical diameter of ICRS is 5.0 mm (AFR5) (the flat basis width is 0.6 mm) or 6.0 mm (AFR6) (the flat basis width is 0.8 mm), with variable thickness (0.15 mm to 0.30 mm with 0.05 mm steps) and arc lengths (90, 120, 150, and 210 degrees). The Ferrara-type ICRS were implanted following the nomogram used in previous studies [19–22]. The same surgeon (JFA) performed all the procedures using topical anaesthesia and following the standard procedure as previously described [19–22].

Postoperative treatment consisted of the combination of antibiotic (tobramycin, 3 mg/mL) and steroid (dexamethasone, 1 mg/mL) eye drops (Tobradex, Alcon Laboratories Inc., Fort Worth, Texas, USA) administered three times daily for 2 weeks.

Cataract extraction with IOL implantation was performed 6 months after ICRS implantation. The IOL implanted was an extended range of vision IOL (Tecnis Symfony, Abbott Alb Inc.). The posterior surface of this IOL incorporates a 5.5 mm diffractive area which is aimed at compensating the eye's chromatic aberration and increasing the depth of focus. All surgeries in this study were performed by an experienced surgeon (JFA) using peribulbar anaesthesia and a 2.2 mm to 3.2 mm axis incision in order to reduce the preexisting astigmatism. Phacoemulsification was performed with the INFINITI vision system (Alcon Laboratories, Fort Worth, Texas). Phacoemulsification was followed by irrigation and aspiration of the cortex and IOL implantation in the capsular bag using the injector developed for the specific IOL.

Axial length and anterior segment size were measured with the IOLMaster biometer (Carl Zeiss Meditec, Germany, software version 5.4). We chose the SRK/T formula for IOL power calculation. In order to reduce the astigmatism, axis incisions were performed. In eyes with astigmatism less than 1.25 D, one axis incision (2.2 mm) was performed on the steepest meridian. In eyes with astigmatism higher than 1.50 D, two opposite axis incisions (3.2 mm) were created on the steepest meridian, as what previous authors have done in phacoemulsification [14, 15]. All incisions were performed with a bevel-up steel blade (Equipsa S.A., Madrid, Spain).

All patients had a complete ophthalmologic examination preoperatively, 6 months after ICRS implantation (before cataract surgery), and 6 months after IOL implantation.

The clinical measurement taken primarily included corneal topography (Sirius, CSO, Italy), anterior segment optical coherence tomography (Visante Zeiss Meditec, Germany), uncorrected (UDVA) and best-corrected (CDVA) distance visual acuity (ETDRS charts), and manifest and cycloplegic refractions. The Thibos and Horner [23] power method was used to assess presurgery and postsurgery refraction findings. Furthermore, through-focus monocular logMAR visual acuity (defocus curve) was also measured 6 months after IOL implantation. Patients observed a distance ETDRS chart through lenses that increased from +2.00 to −5.00 D in 0.50 D steps. The magnitude of depth of focus depends on how it is defined, and for our study, we used the criterion that depth of focus is the range of focussing error for which the visual acuity does not decrease below two lines of CDVA.

Data analysis was performed using SPSS for Windows, version 14.0 (SPSS Inc., Chicago, IL). Normality was checked by the Kolmogorov-Smirnov test, and a repeated measures analysis of variance (ANOVA) was performed to compare outcomes. Differences were considered to be statistically significant when the *P* value was <0.01.

## 3. Results

This study comprised 17 eyes of 11 patients with a mean age of 59 ± 12.8 years old.  Table 1 shows the patient's demographics.

**Figure 1:**
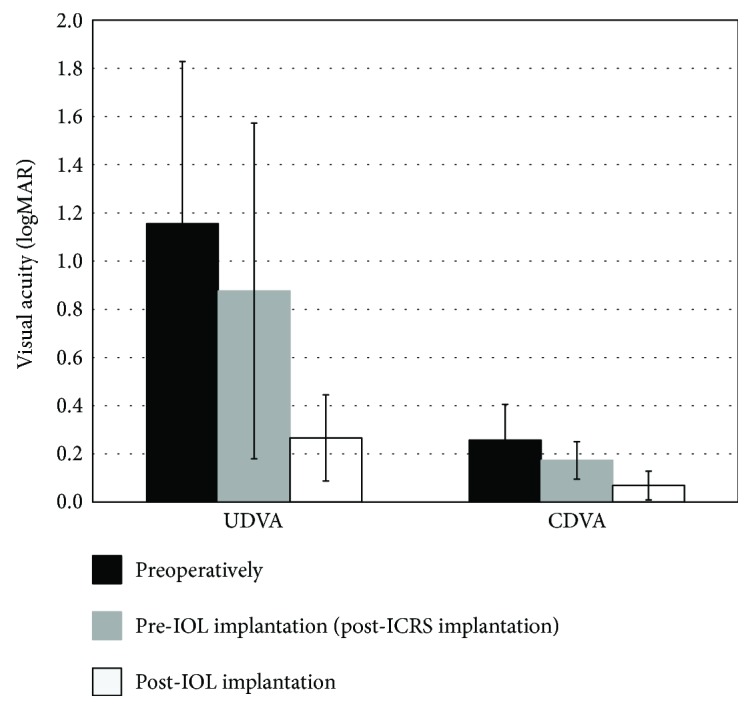
The uncorrected distance visual acuity (UDVA) and corrected distance visual acuity (CDVA) before surgery, 6 months after intrastromal corneal ring segments (ICRS) implantation, and 6 months after intraocular lens (IOL) implantation (efficacy).

Figure 1 shows the efficacy of the ICRS and IOL procedures. UDVA and CDVA (logMAR scale) rose significantly after both surgeries (*P* < 0.0001). The mean UDVA (logMAR) varied from the preoperative 1.15 ± 0.67 to 0.88 ± 0.69 six months after ICRS implantation (before IOL implantation) (*P* = 0.005) and 0.27 ± 0.18 six months after IOL implantation (*P* < 0.0001). The mean CDVA was 0.26 ± 0.15 (logMAR) before ICRS implantation, 0.17 ± 0.08 six months after ICRS implantation (*P* = 0.002), and 0.07 ± 0.06 six months after IOL implantation (*P* < 0.0001). The efficacy index (mean postoperative UDVA/mean preoperative CDVA) 6 months after ICRS implantation was 0.50 and 6 months after IOL implantation was 0.85. There were no statistically significant differences between UDVA after the whole procedure (ICRS + IOL implantation) and the preoperative CDVA (*P* = 0.4), which provided an efficacy index of 1.00.

**Figure 2:**
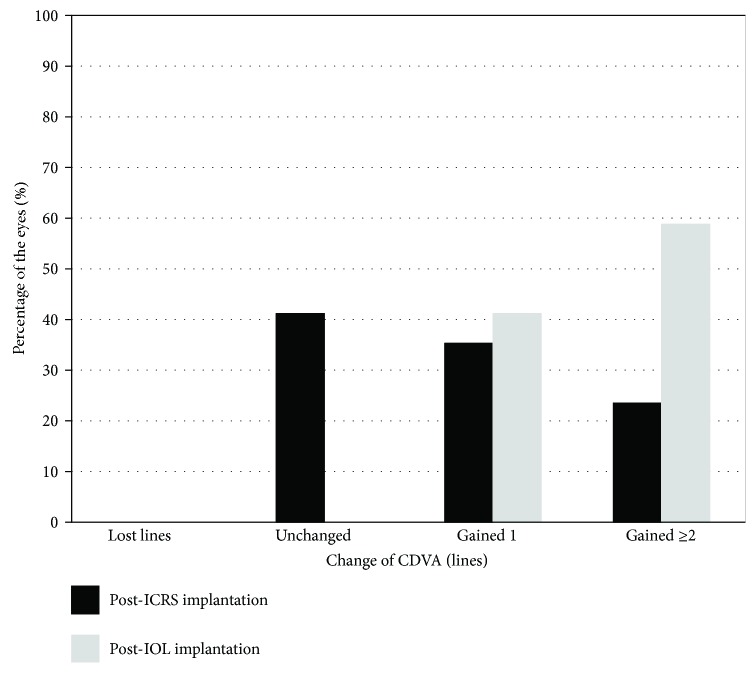
Change in corrected distance visual acuity (CDVA) 6 months after intrastromal corneal ring segments (ICRS) implantation and 6 months after intraocular lens (IOL) implantation (safety).

None of the patients lost lines of CDVA after any of the surgeries (see Figure 2). By six months after ICRS implantation, 7 had no change of CDVA, 6 eyes gained one line, and 4 eyes gained two lines or more. The safety index 6 months after ICRS implantation (ratio of postoperative and preoperative monocular CDVA) was 1.18. By six months after IOL implantation, all eyes gained CDVA, 7 eyes gained one line, and 10 eyes gained two lines or more of CDVA. The safety index 6 months after IOL implantation was 1.17. The safety index of the whole procedure (ICRS + IOL implantation) was 1.26.

**Figure 3:**
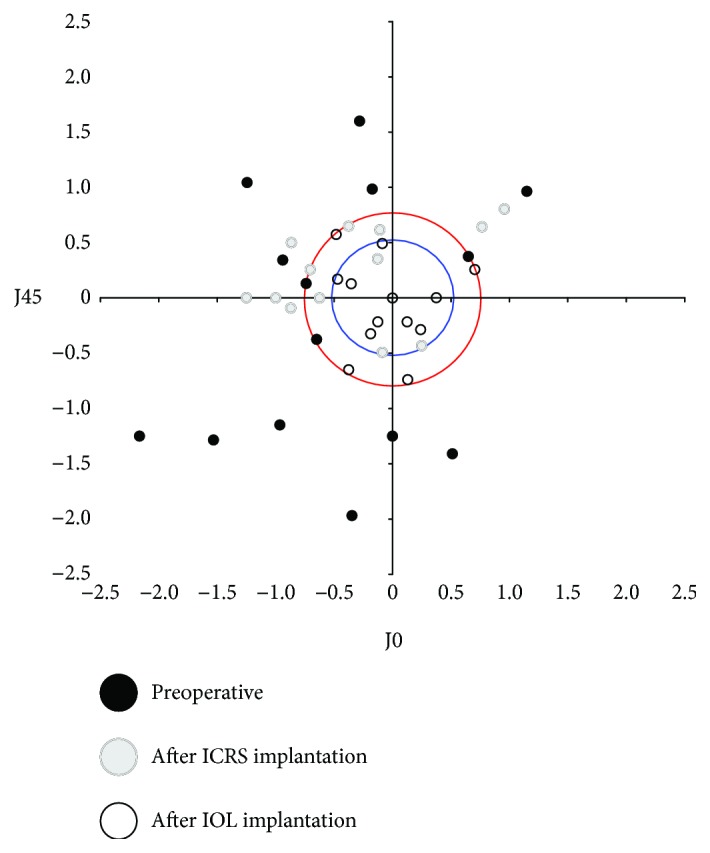
Representation of the astigmatic vector (J0 and J45) before surgery, 6 months after intrastromal corneal ring segments (ICRS) implantation, and 6 months after intraocular lens (IOL) implantation.

Table 2 shows the distribution of manifest refraction error (power vector method) preoperatively, 6 months after ICRS implantation, and 6 months after IOL implantation. There was a large reduction in *M* value (spherical equivalent) and *B* value (blur strength) after surgery (*P* < 0.0001). Six months after IOL implantation, the spherical equivalent was ≤1.00 D in 86.7% of the eyes.  Figure 3 shows the astigmatism component of the power vector represented by a two-dimensional vector (J_0_, J_45_). The origin of the graph (0, 0) represents an eye free of astigmatism. The spread of the post-ICRS implantation data from the origin is more concentrated than the spread of the preoperative data. The spread in the post-ICRS implantation data was converted into a concentrated data set around the origin after IOL implantation. The percentage of eyes with a refractive cylinder ≤ −1.5 D increased from 17.6% preoperatively to 100% six months after IOL implantation (Figure 3, red circle), while the percentage of eyes with a refractive cylinder ≤ −1.0 D varied from 0% to 76.5% (13 eyes) (Figure 3, blue circle).

**Figure 4:**
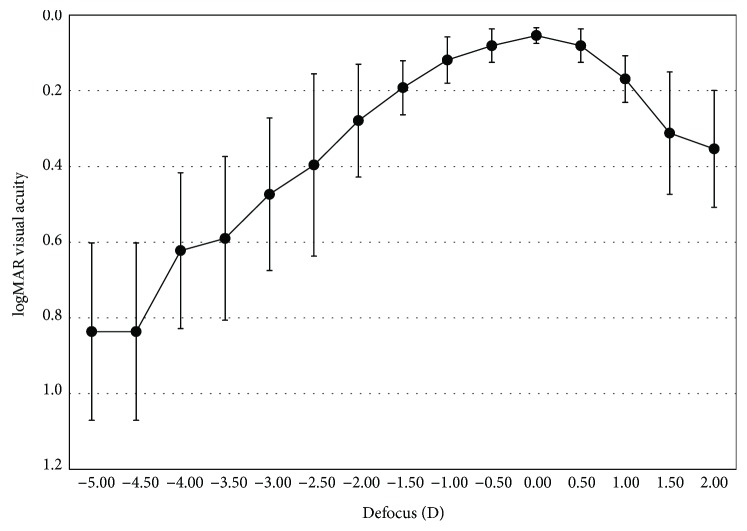
Mean high-contrast monocular logMAR acuity with best correction for distance as a function of the lens defocus (D).

Figure 4 shows the defocus curve for each group separately. The magnitude of depth of focus was 2.60 ± 1.02 D. There were no statistically significant differences in visual acuity for a defocus range from +0.50 D to −0.50 D (*P* > 0.1)

## 4. Discussion

Earlier studies [3–15] have assessed several alternatives for replacement of the lens in keratoconic patients. The most studied approach has been the replacement of the lens by a toric IOL. The first three studies [3–5] were case reports, which showed encouraging results. Subsequent case series studies [6–10] (from 12 to 23 eyes) reported a significant improvement in UDVA, CDVA, and refractive error. The visual and refractive outcomes of multifocal toric IOL have been also evaluated. Montano et al. [11] described two cases, a “forme fruste” keratoconus and a stable keratoconus. Farideh et al. [12] evaluated the clinical results of toric intraocular trifocal IOL in 10 eyes (5 patients) with mild keratoconus. Both studies concluded that multifocal toric IOL provides satisfactory results in mild and stable keratoconus.

Despite these encouraging outcomes for visual quality restoring in patients with cataract and keratoconus, all these approaches should face two challenges: the first challenge is that the corneal abnormalities may lessen the optimal restoration of the visual quality and the second challenge is the IOL power estimation. Regarding the first challenge, a previous study [8] reported that patients with more regular corneas obtained higher improvement of UDVA after surgery. A previous study [14] from our research group reported the visual and refractive outcomes of a combined procedure (ICRS + monofocal IOL implantation). This sequential procedure is aimed at providing the higher level of visual rehabilitation in patients with keratoconus and cataract by improving the corneal shape and removing cataract and refractive error. In this previous study, both the UDVA and CDVA improved after each procedure. The UDVA and CDVA (logMAR scale) six months after IOL implantation were 0.44 ± 0.29 and 0.11 ± 0.16, respectively. In the current study, the UDVA and CDVA also improved after each procedure (ICRS implantation and an extended range of vision IOL implantation). The CDVA improvement after the whole procedure was greater in the previous study. However, in the current study the UDVA improvement was greater than those reported in the previous one (from 1.15 ± 0.67 (logMAR) to 0.27 ± 0.18 and from 1.08 ± 0.24 to 0.44 ± 0.29, resp.). The difference in the spherical equivalent between the two studies after the whole procedure was less than a quarter of dioptre. The difference in the CDVA and UDVA results can be attributed to the IOL implanted (monofocal versus extended range of vision IOL). A monofocal IOL provides a better CDVA than an extended range of vision IOL; while, as explained below, the residual refractive errors can be better tolerated with an extended range of vision IOLs.

Choosing the IOL power may be a challenge in keratoconic patients. Leccisotti [24] reported that refractive exchange in keratoconic eyes is a predictable procedure to correct myopia. However, 32% of the cases required an IOL exchange due to inaccurate IOL power calculation. Thebpatiphat et al. [2] compared the SRKI, SRKII, and SRK/T IOL formulas in patients with keratoconus and suggested that the SRKII formula might provide the most accurate IOL power in patients with mild keratoconus. However, in moderate and severe keratoconus, IOL calculations were less accurate and no differences in calculation formulas were found. A source of error for IOL power calculation in keratoconic patients is the determination of the optical power of the cornea. Usually, the power of cornea is estimated by considering only the radius of the anterior surface and a simulated refractive keratometric index. This estimation could lead to inaccuracies in the calculation of total corneal power in keratoconic eyes, where both the anterior and posterior surfaces of the cornea can be affected. ICRS implantation before cataract surgery could help to regularize the corneal shape and consequently minimize the inaccuracies in the determination of optical power of the cornea. An earlier study [14] found that the spherical equivalent after sequential implantation of the Ferrara-type ICRS and IOL implantation was −0.82 ± 0.91 D. The refractive outcome results of the current study are in accordance with the previous one. Six months after IOL implantation, the mean spherical equivalent was −0.59 ± 0.80 and 86.7% of eyes had a spherical equivalent ≤ 1.00 D. In addition to the predictability of the refractive outcomes, an important aspect is the impact of the residual refractive error on the visual acuity outcomes, in other words, the tolerance to defocus. In the current study, an extended range of vision IOL was implanted which is aimed at increasing the tolerance to defocus. Analysing the defocus curve shown in Figure 4, there were no statistically significant differences in visual acuity for a defocus range from +0.50 D to −0.50 D. These findings could suggest that some residual refractive errors can be tolerated after this combined procedure (ICRS + an extended range of vision IOL implantation). In addition, the magnitude of depth of focus was 2.60 ± 1.02 D, which provide an optimal visual acuity at intermediate distance (Figure 4).

The success of this sequential procedure requires knowledge of the risk of progression of keratoconus, because the progression of keratoconus can lead to refraction change and it could be a problem after IOL implantation. A previous study from our research group [14] showed that sequential Ferrara-type ICRS and IOL implantation provides stable visual and refractive outcomes. In the current study, the only difference was the IOL implanted; hence, it seems logical to think that the visual and refractive outcomes will be stable too. However, further long-term studies should be carried out to confirm this hypothesis and to assess whether small corneal changes could have more impact after an extended range of vision IOL implantation than a monofocal IOL implantation.

In conclusion, our outcomes suggest that sequential Ferrara-type ICRS and extended range of vision IOL implantation provides good visual and refractive outcomes, being an effective, safe, and predictable procedure for the treatment of selected cases of patients with keratoconus and cataract. In addition, this approach provides an increase of tolerance to defocus.

## Figures and Tables

**Table 1:** Patient demographics. Age, pre-ICRS implantation manifest refraction (spherical equivalent (SE), refractive sphere and cylinder) and prekeratometry (K) readings shown as mean ± standard deviation (SD) and range.

Characteristic	Value
Eyes (*n*)	17
Age (years)	59 ± 12.8
Mean SE (D) Range	−5.35 ± 5.09 (+2.50 to −14.00)
Mean refractive sphere (D) Range	−3.97 ± 4.98 (+3.50 to −13.00)
Mean refractive cylinder (D) Range	−2.77 ± 1.04 (−1.50 to −5.00)
Mean minimum K (D) Range	47.06 ± 3.71 (42.5 to 55.5)
Mean maximum K (D) Range	48.79 ± 3.69 (45 to 57.75)

**Table 2:** Summary of distribution of manifest refractive errors before surgery, 6 months after ICRS implantation, and 6 months after IOL implantation, following the power vector method.

	Preoperatively	After ICRS implantation	After IOL implantation	*P value*
M	−5.35 ± 5.09^∗^	−3.45 ± 3.88^∗∗^	−0.59 ± 0.80	<0.0001
J_0_	−0.40 ± 0.92^∗^	−0.32 ± 0.63^∗∗^	−0.07 ± 0.40	*P* = 0.009
J_45_	0.17 ± 1.13	0.23 ± 0.40^∗∗^	−0.09 ± 0.57	*P* = 0.02
B	6.28 ± 4.08^∗^	4.17 ± 3.15^∗∗^	0.94 ± 0.74	<0.0001

Data are shown as mean ± standard deviation. Manifest refraction in conventional script notation (S (sphere), C (cylinder) × *ϕ* (axis)), were converted to power vector coordinates and overall strength blur by the following formulas: M = S + C/2; J_0_ = (−C/2) cos (2*ϕ*); J_45_ = (−C/2) sin (2*ϕ*); B = (M^2^ + J_0_^2^ + J_45_^2^)^1/2^. ^∗^Statistically significant between before Keraring ICRS insertion and after IOLs implantation. ^∗∗^Statistically significant between after Keraring ICRS implantation and after IOLs implantation.
